# Outcome of surgical repair of Pectus Excavatum in adults

**DOI:** 10.1186/s13019-017-0635-z

**Published:** 2017-08-29

**Authors:** Ayman M. Shaalan, Ibrahim Kasb, Eman E. Elwakeel, Yusra A. Elkamali

**Affiliations:** 1 Cardiothoracic Surgery Department, Benha University, Benha, Egypt; 2 Dallah Hospital, Cardiac Center, Riyadh, Saudi Arabia; 3 Anatomy and Embryology Department, Benha University, Benha, Egypt; 4 Statistics Department, Riyadh Colleges of Dentistry and Pharmacy, Riyadh, Saudi Arabia

**Keywords:** Pectus Excavatum, Surgical repair, Surgical outcome, Adults

## Abstract

**Background:**

Pectus Excavatum (PEx) is the most common congenital chest wall deformity, accounting for over 90% of all chest wall deformities. Surgical correction is recommended because severe PEx can affect the physical and psychological development of patients. The aim of our study was to assess the impact of surgical repair of Pectus Excavatum in adults during hospital course and results after 1 year.

**Methods:**

Prospective study was carried out on 86 adult patients aged ≥ 15 years, 52 males and 34 females (mean age was 26 ± 1.5 years). All cases were divided into two groups, group I: (15–25 years old) and group II: (> 25 years old). Preoperative, operative, and postoperative data were reviewed. Statistical analysis was performed.

**Results:**

Statistical analyses revealed significant improvement postoperatively of cosmetic satisfaction (*P*-value < 0.0001), pain (*P*-value =0.0003), exertional dyspnea (*p*-value <0.05) and exercise tolerance. The degree of chest compression was significantly improved after surgical correction within 12 months and the estimated measurement postoperatively of Haller Index showed significant reduction (*p*-value <0.001). Patient satisfaction postoperatively was excellent in 77.9% of all cases.

**Conclusion:**

Surgical correction of Pectus Excavatum using open technique in adults had excellent post-operative outcome in the short term follow up that encourage performing the procedure for all cases. Long term results need longer period for follow up. Etiology and predisposing factors still need further research.

## Background

**Fig. 1:**
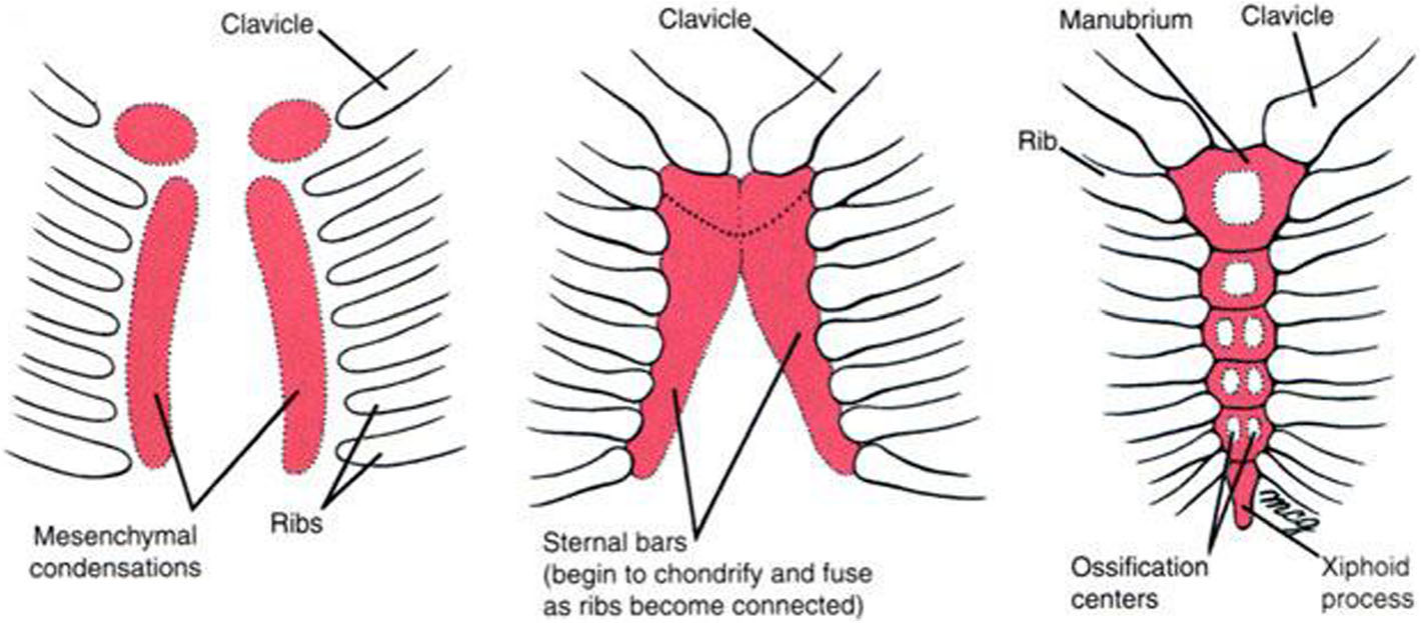
Development of the sternum

Pectus Excavatum (PEx) is the most common congenital chest wall deformity, accounting for over 90% of all chest wall deformities, with an incidence rate of approximately 0.1%, and a male to female ratio of 4:1 [1]. Within this congenital chest wall deformity, several ribs and the sternum grow abnormally, producing caved-in appearance in the anterior chest wall. The sternum develops from the somatic mesoderm in the ventral body wall. Two sternal parts are formed on either side of the midline and these later fuses to form the cartilaginous model of the manubrium, body of the sternum and the xiphoid process. Centers of ossification appear cephalo-caudally before birth except in the xiphoid process which appears during the 3rd year of childhood (Fig. 1). Ossification at the lowest segment begins shortly after birth [2].

Surgical correction is recommended because severe PEx can affect the physical and psychological development of patients [1]. Cartilage repair is typically slow and incomplete. However, an exception to this observation can be found in the costal cartilages, where complete repair has been reported in humans. Full replacement of the resected cartilage occurs quickly (within 1 to 2 months) and properly differentiates, but that repair occurs only in the presence of the perichondrium [3].

This deformity varies in extent, may be short or long segment and may be symmetrical or asymmetrical. The degree of internal compression is variable and can be graded by HI (Haller Index). HI (maximal transverse diameter/narrowest AP length of chest) is used to assess severity of incursion of the sternum into the mediastinum. Normal Haller Index is 2.5. Familial predisposition has been observed and nearly half of the patients have relatives with various skeletal alterations [4, 5].

Progression may be particularly evident during adolescence; this information is unfamiliar to a lot of pediatricians, who erroneously inform younger patients that this condition will resolve spontaneously with growth. At the time of puberty, when patients grow rapidly, such deformity often abruptly accelerates; a mild defect may quickly turn into severe form. Surgical repair of PEx in most of cases has a cosmetic indication with mild symptoms [6].

Routine early repair of Pectus Excavatum in patients older than 3 years of age is safe and effective as the chest wall is more malleable. Despite literatures recommended delaying the repair of Pectus deformity until after puberty to obtain more definitive and long-lasting repair [7]. There were limited studies regarding open surgical repair in adults, because of that our aim was to assess the impact of surgical repair of Pectus Excavatum in adults during hospital course and results after 1 year.

## Methods

This study was prospective study, carried out on 86 adult patients aged ≥ 15 years. It was done in three tertiary centers in developing countries between 2010 to 2015 and approved by the Ethical Committee of the Centers. Informed, written consent was obtained from each patient including a clause for using the images and data for research purposes.

The cases were divided into two groups, group I: (≥ 15–25 years old) and group II: (> 25 years old). First group represented the early adolescence, in this age noticed the decrease in rate of growth of ribs, while second group represented lower rate of rib growth and more rigid skeleton. All cases were evaluated clinically. Routine investigations were done as lab tests, EKG, echocardiography, chest X-ray. Plain CT chest was done for diagnosis and calculation of Haller Index to assess the severity preoperatively and the improvement postoperative. All patients were graded according to the Haller Index (HI) into: mild (HI <3.2), moderate (HI 3.2–3.5), severe (HI 3.6–6.0), extremely severe (HI > 6.0). Multiple grading were used in our study to differentiate the grade of compression in each patient with related symptoms and to clarify the degree of improvement after repair.Also to follow the classic HI grading. Pain scoring system (numerical graded from 1 to 10) was considered for pain assessment pre and post operatively. Dyspnea and easy fatigability symptoms were evaluated by the referring physicians as these symptoms were correlated with NYHA grading system (New York Heart Association functional classification). Operative time, ICU, hospital stay were calculated. Follow up for operative, early and late post-operative course were evaluated in outpatient clinic visits.

### Inclusion criteria

All patients with isolated PEx aged ≥ 15 years old, those with HI moderate, severe or extremely severe. Who fulfill the indications: (1) CT chest with moderate HI > 3.25, (2) bony deformity distressing the patient psychologically, (3) Progressive bone abnormality, (4) bony deformity with progressive symptoms secondary to compression either cardiac or pulmonary with positive finding in EKG, Echocardiography or pulmonary function study.

### Exclusion criteria

Patients below 15 years, asymptomatic cases and those with mild degree. Patients with cardiac disease congenital or acquired (no cases had heart failure) or cases who had pulmonary diseases as (bronchiectasis or cystic lung lesion and emphysematous lung). Also cases with neuromuscular illness affecting breathing. All cases that required other surgical procedures simultaneously either thoracic or cardiac cases were excluded. Recurrent PEx after previous repair.

### Surgical procedure

The patients fill in the consent for open surgical repair and for insertion of thoracic epidural cannula for analgesia post-operative. Patient was in supine position under general anesthesia with full monitoring. Before induction of anesthesia, the site of deformity was demarcated from the 3 aspects, vertical diameter, transverse diameter and the deepest point of deformity.

**Fig. 2:**
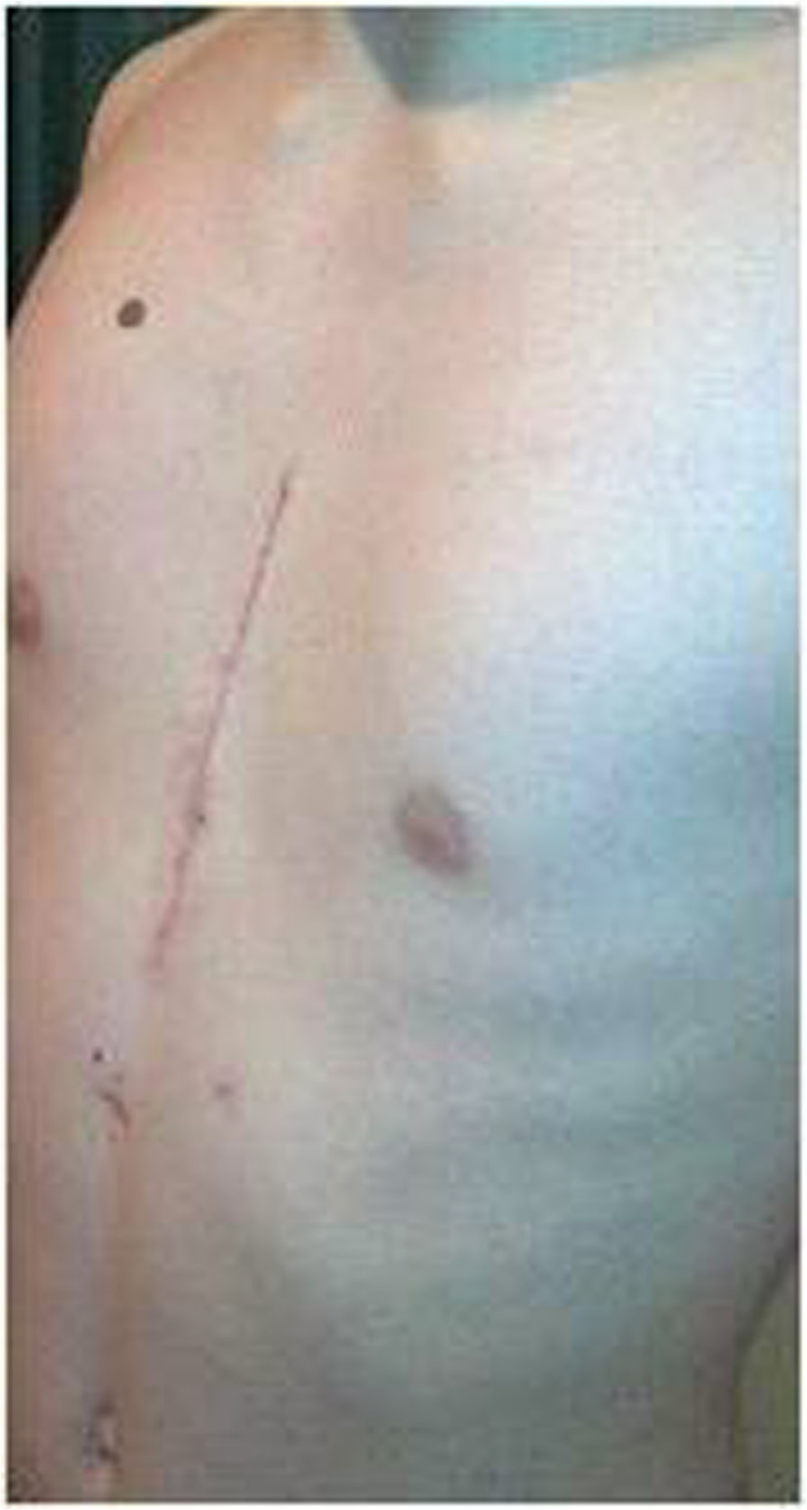
Post-operative repair midline longitudinal incision

Longitudinal incision was done above the point of angulation till the xyphoid process (Fig. 2). Muscle Splitting was done between the two pectoral muscles then undermining was done till the costochondral junction. This made more exposure for all deformed cartilages. Resection of all deformed cartilages from sternum till the costal end after dissection from perichondrium was applied, leaving perichondrium intact to allow re-growth of the cartilage in newly positioned sternum. The difficulty was found in older group of patients as the perichondrium was more adherent, needed careful dissection and undermining using periosteal elevator and Doyen rib elevator passed around the cartilage taking more time.Freeing the sternum at the xyphoid end was essential. At the site of maximum angulation, V-shaped osteotomy was done to allow free upward movement of the lower part of the sternum to its new position. Steel metal bar was prepared according to the dimension of thoracic cage and the level of maximum deformity and then the metal bar was placed behind the sternum for support and was fixed bilaterally at anterior axillary line. Chest drains in both pleurae were inserted in cases where both pleurae were opened.J-vac drain (Jackson -pratt) or hemovac drain was inserted retro muscular for drainage of any discharge. The wound was closed in layers. Lastly all cases were extubated postoperatively. They were shifted to regular room unless there was a need for ICU care. Cefazolin IV doses were given in the first 48 h, unless there was any other indication to continue or to change antibiotics according to culture and sensitivity in cases of wound infection. Epidural analgesia was used for 2 days postoperatively for patients who accepted its insertion. Patients were encouraged to ambulate and to use incentive spirometer.

**Fig. 3:**
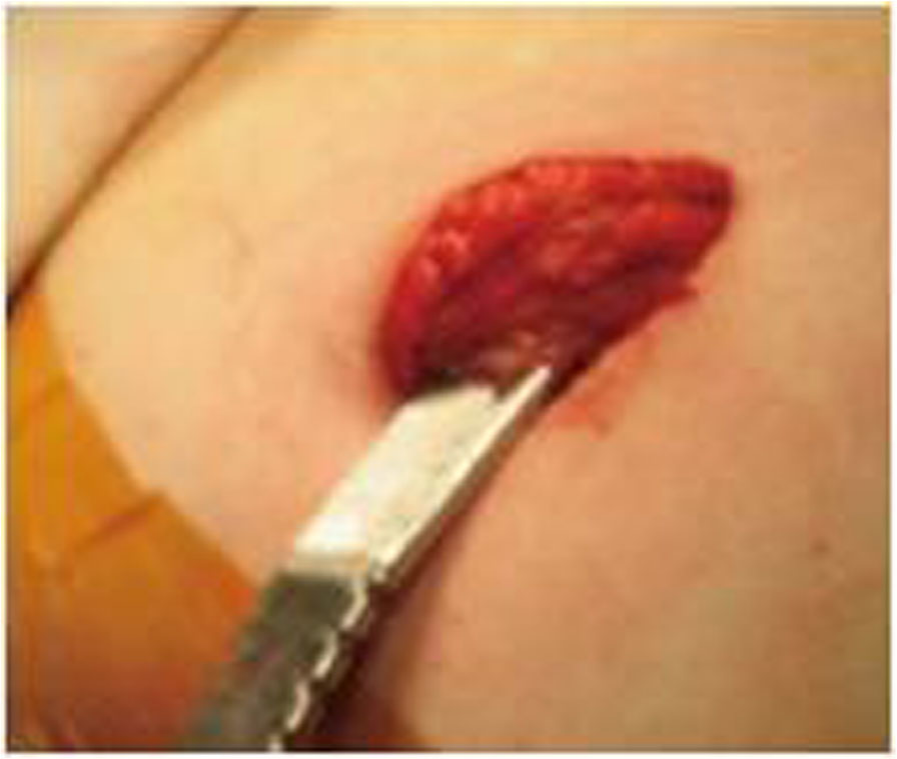
Metal bar removal by small lateral incision

Most of the patients were discharged home within 4–7 days after surgery according to the following criteria: hemodynamic stability, dry clean wound, stable sternum, chest x-ray, no significant symptoms and pain score less than 4/10 controlled with NSAID. Follow up visits were arranged on regular bases. Patients were instructed not to do aggressive movement e.g. bend and rotate their bodies in the first 2 months. All patients instructed to take analgesics if there was pain, to avoid carrying heavy weight and to avoid strenuous exercise to give chance for better healing in the new position. Regular activities were permitted thereafter. Regarding any discharge or wound problem, they were instructed to come immediately for evaluation. Regular visits were arranged at 2 weeks, 2 months, 8 months and 1 year to assess the patient clinically and radiological. CT chest (computed tomography) was done at 12th month visit. Metal bars were planned to be removed after 12 months from surgery unless there was any concern (Fig. 3).

Patient satisfaction was assessed by the surgeons in the clinic through radiological and clinical evaluation after 12 months according to: (I) Chest X-ray showed no sternum depression; (II) the morphology of chest wall was symmetry without depression; (III) The patient and their families were satisfied; (IV) The thorax appeared full with good extension and elasticity. The outcome was considered excellent for 4, good for 3, fair for 2 and poor for less than 1 [1].

### Statistical analysis

All data were collected, organized, tabulated, and statistically analyzed using SPSS software statistical computer package version 13 (SPSS, Chicago, Illinois, USA). For quantitative data, the range, mean, and standard deviation were calculated. Correlation between variables was evaluated. Chi-Square (χ2) test was used to compare proportions between group I and group II. Significance was adopted at *P* < 0.05 for interpretation of results of tests of significance.

## Results

This study included 86 cases, 52 male (60.5%) and 34 female (39.5%). Their ages ranged from 15 to 47 years with the mean age (26±1.5) years. These cases were divided into two groups, group I: (15-25 years) and group II: (> 25 years) to assess the surgical outcome in adult cases and to compare between both groups to detect any age related difference. There were positive family history, connective tissue disease and weak mesenchyme in 23 % of cases. Among our cases there were 6 cases been brothers from 3 families with different grades of deformity that required surgical repair. The clinical evaluation of all cases showed, the mean weight (68 ± 1.5) Kg, height (168 ± 9) cm. There were different clinical presentations with different complaints and combined symptoms between both groups and even in the same group. These symptoms in the form of pain, palpitation, exertional dyspnea and easy fatigability showed no significance differences between both groups. Also psychological complain secondary to chest deformity was 25% in group I and 91.3% in group II. There was numerical difference between both groups with significant difference regarding cosmetic unsatisfaction (Chi-square test = 38.884, *P*-value < 0.0001) (Table 1).

**Table 1:** Clinical presentations of cases in both groups

Manifestations	Group I (15–25) years (*n* = 40)		Group II > 25 years (*n* = 46)		*χ*2 *test*	*P*-value
	Number	%	Number	%		
Palpitation	12	30%	9	19.6%	1.23	0.26 NS
Easy fatigability	34	85%	32	69.6%	2.8	0.09 NS
Excertional dyspnea	28	70%	23	50%	3.5	0.06 NS
Pain	40	100%	46%	100%		NS
Cosmetic satisfaction	10	25%	42	91.3%	38.884	*P* < 0.0001*

*NS* non significant *p*-value

*High Significant *P*-value

Exertional dyspnea was significant complain in most of the cases with variable grades in both groups. Preoperatively dyspnea functional grades in group I were 15% in grade IV, 32.5% in grade III and 22.5% in grade II, while in group II preoperatively, dyspnea were 8.7% in grade IV, 28.26% in grade III and 13.04% in grade II (Table 2).

**Table 2:** Dyspnea functional status of patients pre-operatively and post operatively for both groups

Grades of dyspnea	Group I (15–30) years (*n* = 40)					Group II (> 30 years (*n* = 46)				
	Pre-operative		Post-operative		*p*-value	Pre-operative		Post-operative		*p*-value
	N	%	N	%		N	%	N	%	
I	12	30	16	40%	0.23	23	50%	32	69.6%	0.11
II	9	22.5	24	60%	0.0035*	6	13.04%	14	30.4%	0.03*
III	13	32.5	0	0%	0.0002*	13	28.26%	0	0%	0.0003*
IV	6	15	0	0%	0.005*	4	8.7%	0	0%	0.0001*
Total	40	100%	40	100%		46	100%	46	100%	

*Significant *P*-value

Regarding pain was the most significant symptom in all cases; as numerical pain score preoperative was ranged between score 4–6/10 among all cases of both groups (Table 3).

**Table 3:** Pain score pre-operative and post-operative

Pain Score	0	1	2	3	4	5	6	7	8	9	10
Pre-operative	0 0%	0 0%	0 0%	0 0%	8 9.3%	28 32.5%	34 39.5%	16 18.6%	0 0%	0 0%	0 0%
Post- operative	0 0%	66 76.7%	14 16.2%	0 0%	6 6.9%	0 0%	0 0%	0 0%	0 0%	0 0%	0 0%
*p*-value	No	0.0003*	0.003		0.29 NS	0.0025*	0.0003*	0.0003*			
*χ*2 *test*		16	4		0.55	6	7.5	4.4			

*High Significant *P*-value

In our study, there were 21 cases preoperatively complained of palpitation documented by EKG, 12 cases in group I (30%) and 9 cases in group II (19.6%) with no significant difference between two age groups (Chi-square test = 1.23, *P*-value = 0.26) (Table 1). Their diagnosis by EKG was AF in 12.8% of all cases and supraventricular tachycardia in 11.6% of cases from both groups, while 75.6% showed sinus rhythm (Table 4).

**Table 4:** Rhythm difference pre-operative and post- operative

Rhythm	Group I		Statistical test		Group 2		Statistical test	
	Pre-operative	Post-operative	*χ*2	*P*-value	Pre-operative	Post-operative	*χ*2 *test*	*P*-value
AF	8 (20%)	0%	3.16	0.002*	3 (6.5%)	2 (4.3%)	0.455	0.33
Supraventricular tachycardia	4 (10%)	0%	2.11	0.02*	6 (13.04%)	0 (0%)	2.62	0.006*
Sinus rhythm	28 (70%)	40 (100%)	5.72	0.0003*	37 (80.4%)	44 (95.7%)	0.88	0.19
Total	40 (100%)				40 (100%)	46 (100%)		

*Significant *P*-value

Chest x-ray and plain CT chest were used routinely preoperative and in postoperative follow up. The degree of chest compression was estimated and graded preoperatively according to the Haller Index (HI), which were classified into mild, moderate, severe and extremely severe. HI preoperatively was ranged from minimum 3.5, maximum 12.7, mean HI (7.5 ± 0.8), with variable degrees of manifestations as easy fatigability, dyspnea, palpitation, pain and cosmetic unsatisfaction (Table 1).

Regarding the grades of HI preoperatively for both groups, 40 cases (46.5%) were extremely severe grade, 38 cases (44.2%) were severe, 8 cases (9.3%) were moderate grade (Table 5).

**Table 5:** Haller Index (HI) pre operatively and post operatively

Grades of HI	Pre-operative	Post-operative	*χ*2 test	*P*-value
Mild < 3.2	0 (0%)	83 (96.5%)	48.7	0.00025*
Moderate (3.2–3.5)	8 (9.3%)	3 (3.4%)	1.6	0.06
Severe (3.6–6)	38 (44.2%)	0 (0%)	8.3	0.0003*
Extremely severe > 6	40 (46.5%)	0 (0%)	8.6	0.00025*

*High Significant *P*-value

In this study about 73.3% of cases accepted insertion of thoracic epidural anesthesia, while 26.7% of cases accepted paravertebral nerve block using Marcaine which was used intraoperative to decrease pain early postoperative. Number of costal cartilage resected was variable according to the degree and extent of deformity.

Regarding the metal bar, the length of it and its configuration was designed according to point of maximum angulation, intra operative assessment and flexibility of the skeleton. No cases were documented to have metal allergy.

Regarding operative time, there was significant difference between both groups as it was (4.29 ± 0.25) hours in group II, it was (2.25 ± 0.75) hours in group I and the mean duration for both groups was (3.27 ± 0.5) hours.The mean ICU stay was (22.5 ± 13) hours in group II, while it was (10.5 ± 3) hours in group I. The mean hospital stay was (6.9 ± 2.5) days in group II and (3.5 ± 0.5) days in group I, which proved better operative and hospital stay in group I than group II (Table 6).

**Table 6:** Post-operative data and hospital course

Variables	Group I (*n* = 40)	Group II (*n* = 46)	*χ*2 test	*p-value*
Operation time/h	2.25 ± 0.75	4.29 ± 0.25	0.0025	< 0.05
ICU stay/h	10.5 ± 3	22.5 ± 13	0.001	< 0.001
Hospital stay/day	3.5 ± 0.5	6.9 ± 2.5	0.002	< 0.005
Satisfaction Excellent	31 (77.5%)	36 (78.2%)	0.006	0.94 NS
Satisfaction Good	7 (17.5%)	8 (17.4)	0.00	0.99 NS
Satisfaction Fair	2 (5%)	2 (4.3%)	0.023	0.89 NS

*NS* non significant *p*-value

**Fig. 4:**
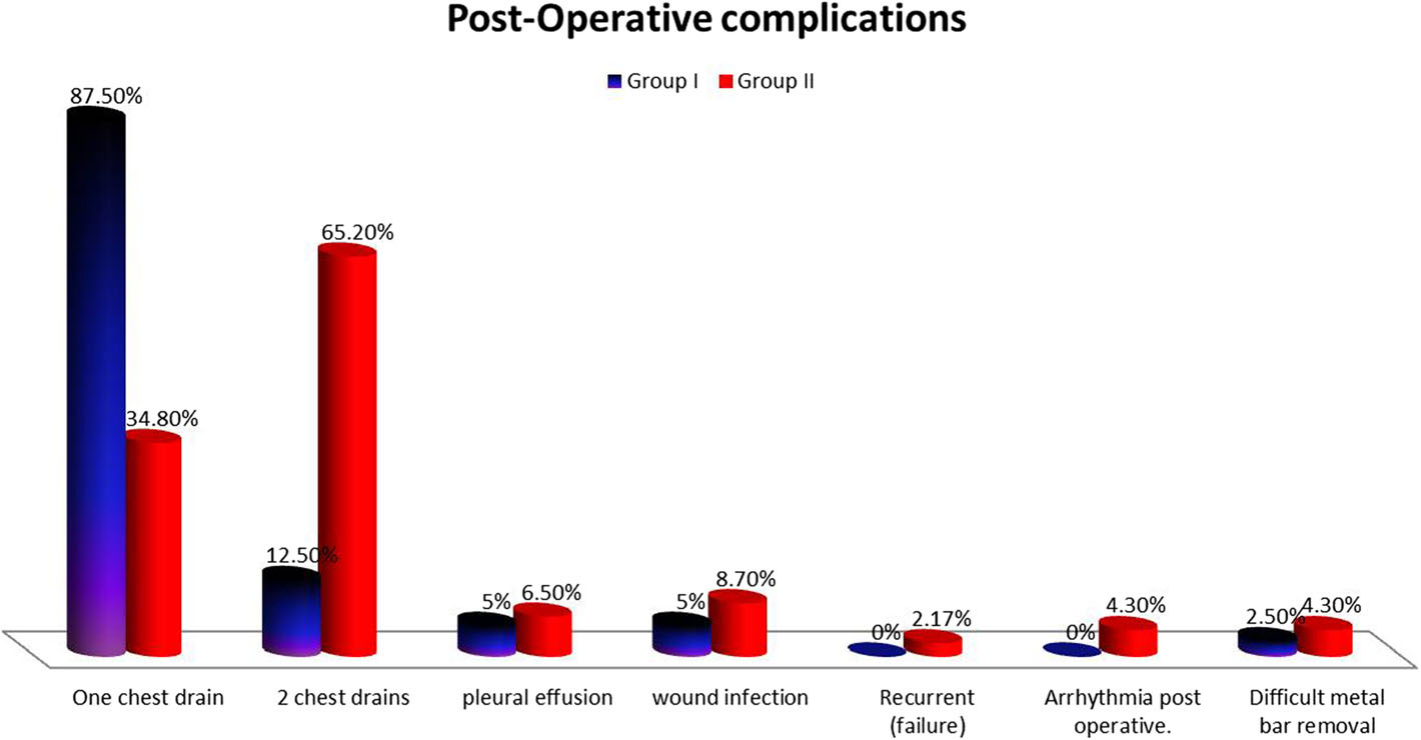
Post-operative complications

There were documented complications noted in both groups without significant differences between them (Fig. 4). In this study, there was no mortality documented in both groups. There was bleeding post-operative in 2 cases from both groups, one case in group II that required re-exploration because of severe bleeding and dropping of hemoglobin to 7 mg/dl with hypotension,bleeding was originated from LIMA and intercostal arteries.The other case was in group II required chest tube insertion and managed conservatively. Pneumothorax after removal of surgical drains was found in 3 cases (3.5%) of both groups and required chest tube insertion with no significant difference in between both groups (Table 7).

**Table 7:** Post-operative complications

Variables	Group I (*n* = 40)	Group II (*n* = 46)	*χ*2 *test*	*p-value*
One chest drain	35 (87.5%)	16 (34.8%)	*24.33*	P < 0.0001*
Two chest drains	5 (12.5%)	30 (65.2%)	*24.33*	P < 0.0001*
Metal allergy	0 (%)	0 (%)	*0*	*NS*
Haemothorax	1 (2.5%)	1 (2.17%)	*0.01*	*0.9 NS*
Pneumothorax	1 (2.5%)	2 (4.3%)	*0.21*	*0.65 NS*
Pleural effusion	2 (5%)	3 (6.5%)	*0.08*	*0.77NS*
Wound infection	2 (5%)	4 (8.7%)	*0.45*	*0.5NS*
Recurrent (failure)	0 (0%)	1 (2.17%)	*0.87*	*0.35NS*
Pain post-operative>2/10	2 (5%)	4 (8.7%)	*0.4*	*0.5NS*
Arrhythmia post-operative.	0 (0%)	2 (4.3%)	*1.74*	*0.19 NS*
Metal bar displacement	1 (2.5%)	0 (0%)	*1.15*	*0.28 NS*
Difficult metal bar removal	1 (2.5%)	2 (4.3%)	*0.21*	*0.65NS*
Mortality	0	0	0	NS

*NS* non significant *p*-value

*High Significant *P*-value

The occurrence of pleural effusion post-operative was noticed in5 cases (5.8%) from both groups, 2 in group I and 3 in group II, required pleural tapping and by pleural analysis were clear no infection. There were wound related complications as keloid postoperative in 3 cases and postoperative wound infection. Infection was manifested in 6 cases (6.9%) in groups, 2 cases in group I and 4 cases in group II. Their wounds were improved with vancomycin except for 2 cases in group II were presented with deep sternal wound infection, one case needed vacuum assisted therapy till healthy granulation tissue appeared then secondary suturing was done, while the second case required wound debridement and cleaning then secondary suturing. One of these cases in group II, also showed infection over the lateral end of metal bar and managed with drainage of collection with frequent dressing till healed with secondary intention. Recurrence of PEx after surgery was noticed in 3 cases (3.4%) in both groups, 1 case in group I and 2 cases in group II (Table 7). In this study difficult metal bar removal occurred in 3 cases and required more extension of the incision with dissection around it (Table 7).

Regarding Post-operative arrhythmia, it was found in 2 cases in group II and diagnosed by EKG to have controlled AF. There was significant reduction of cases who complained of palpitation to 2.3% from the total number of patients and the rest were highly significantly reverted to sinus rhythm. When we compared between AF, supraventricular tachycardia and sinus rhythm data in both groups,the results showed that there were highly significant differences between preoperative and postoperative regarding AF in group I (Chi-square test = 3.16, *P*-value = 0.002), supraventricular tachycardia in group I (Chi-square test = 2.11, *P*-value = 0.02), supraventricular tachycardia in group II (Chi-square test = 2.62, *P*-value = 0.006) and sinus rhythm in group I (Chi-square test = 5.62, *P*-value = 0.0003) (Table 4).

Pain score showed significant improvement postoperatively after 6–12 months, to be score 4/10 in 6 cases, score 2/10 in 14 cases and score 1/10 in 66 cases with highly significant improvement postoperative of pain score to be mostly in score 1/10 (*n* = 66**,** *P*-value =0.0003) These cases with score 4/10 tolerated with NSAID for 2–3 months (Table 4).

**Fig. 5:**
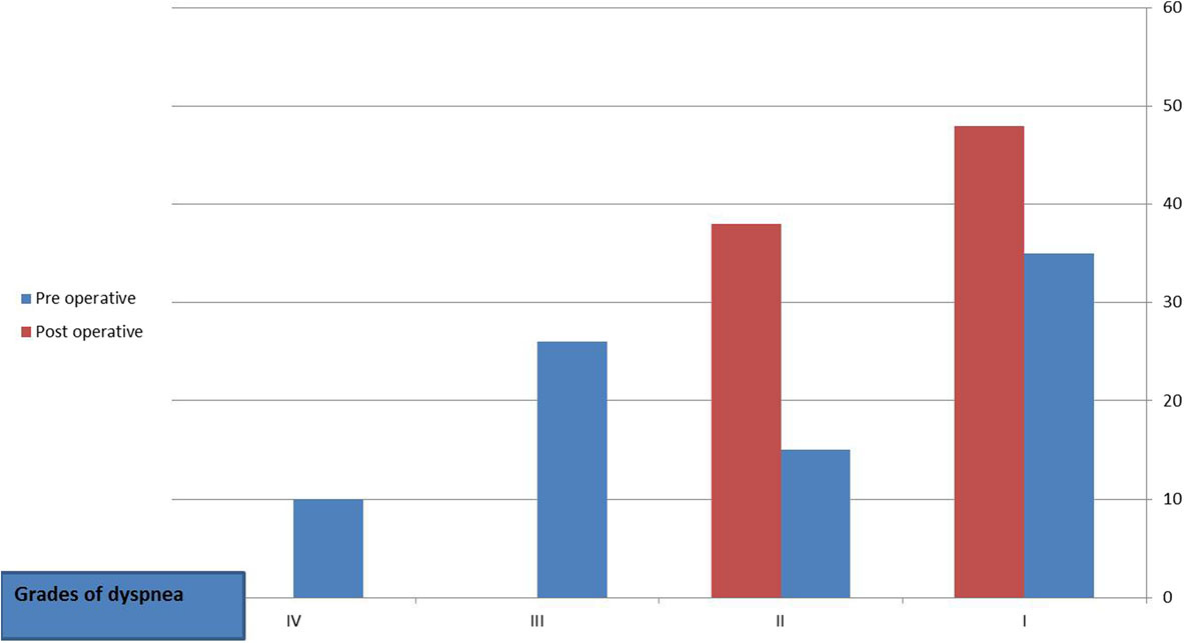
Comparison between degree of dyspnea pre-operative and post-operative

Regarding exertional dyspnea and easy fatigability that were assessed by the referring doctors and correlated to NYHA grading, there was significant improvement in the postoperative follow up (Fig. 5). The dyspnea grade I and II were 16 (40%) and 24 (60%) respectively in group I. also there was remarkable improvement in group II postsurgical repair as 32 (69.6%) in grade I and 14 (30.4%) in grade II. While comparing the grades of dyspnea preoperative and postoperative in each group, we found significant differences in grade III in group I (*p*-value = 0.0002) and grade III in group II (*P* value = 0.0003). Also there were significant differences in grade IV in group I (*p*-value = 0.005) and grade IV in group II (*P* value = 0.0003) (Table 2).

**Fig. 6:**
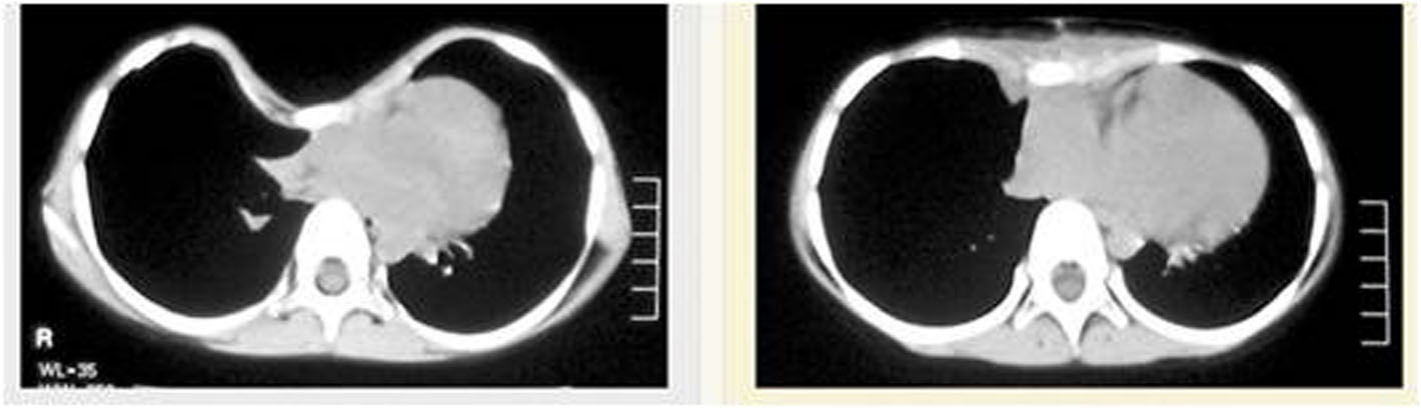
Plain CT chest pre-operative and post-operative follow up

There was significant improvement in chest configuration and degree of compression (Fig. 6). After 12 months of surgery, HI was re-evaluated and showed highly significant improvement to be 83 (96.5%) in mild grade, 3 (3.4%) in moderate grade and 0 (0%) in severe and extremely severe grades. The results showed that there were highly significant differences between Preoperative and Postoperative HI in both groups in mild grade (Chi-square test = 48.7, *P*-value = 0.00025), severe grade (Chi-square test = 8.3, *P*-value = 0.0003) and extremely severe grade (Chi-square test = 8.6, *P*-value = 0.00025) (Table 5). The cases with HI >3.2 postoperative were improved in comparison to preoperative data, as they were graded before repair in the severe and extremely severe groups. Their surgical procedure showed difficulty during cartilage removal, difficulty during v-shaped sternal osteotomy and so rigid skeleton and those who had infection and metal bar dislodgment. They were presented in small percentage 3.4% from both groups (Chi-square test = 1.6, *P*-value = 0.06).

Regarding patient satisfaction, it was assessed by the surgeons in the clinic through radiological and clinical evaluation after 12 months. It was excellent in 67 cases (77.9%), good in 15cases (17.4) % and fair in 4 cases (4.7%) in both groups (Table 6).

## Discussion

Pectus Excavatum is the commonest chest wall deformity seen then pectus Carinatum, bifid sternum and absent sternum.Most of our cases with PEx were referred from other clinics, either complaining from cosmetic unsatisfactory in adult age or lung and cardiac problems secondary to chest compression. This malformation is ranging between 1 in 400 and 1 in 1000 live births. There are many different shapes of chest configurations in Pectus cases. The etiology of Pectus Excavatum is uncertain, but a familial tendency has been found in clinical experience [6]. This is correlated with our study regarding positive family history with similar illness and other connective tissue diseases. Other studies showed that, the etiology remains unclear, but it appears to be polygenetic [8, 9].

Abnormal skeletal growth with inward compression of the sternum upon intra-thoracic structures is the main pathophysiological in this illness. The rigid skeleton in adult cases in comparison to childhood cases, is the challenging during surgical reconstruction to achieve satisfactory results. Other researches said, it is formed as a result from unbalanced growth of the costochondral regions of the anterior chest wall, leading to symmetric and asymmetric anomalies [10].Different surgical techniques developed many years ago, but rigid skeleton in adult cases still the corner stone during repair. Because of that we divided our cases above 15 years as this is the beginning of adolescence with slower rate of rib growth, while above 25 years rib growth became more stationary with more rigid thoracic cage. One of the literatures searched about the limitations of Nuss procedure, they found that better clinical results are achievable in patients less than 12 years of age with a symmetric deformity. In older patients (over 15 years of age) with a rigid chest or with an asymmetric deformity, additional procedures are required to achieve a comprehensive correction of the deformity [11].

CT chest was the golden stander radiological tool in this study to confirm the diagnosis and its grading pre and post operatively. All documented studies about Pectus used HI; also CDI (Cardiac deformity index) can be used as predicting physiological outcomes of the heart performance. The degree of compression on mediastinal structures was directly proportionate to the obvious clinical finding and to the degree of HI [12]. Authors found that, the most common symptoms in this deformity were dyspnea, exercise intolerance, palpitation and chest pain [13].

Arrhythmia was found in a large number of cases before surgical reconstruction, which showed marked improvement after surgery. Literatures proved that, arrhythmia in Pectus Excavatum was documented in two thirds of the patients with lone AF in mild degree of Pectus and 17% have severe form of Pectus compressing right ventricular out flow tract [14]. Others proved that, PEx as a benign condition may lead to serious cardiac events secondary to direct right atrial compression that leads to atrial fibrillation or supraventricular tachycardia [15, 16].

In this study most of the patients were cosmetically unsatisfied and this was the main complain. Patients often have body image embarrassment, which may result in adverse psychological symptoms and lower quality of life [17, 18]. PEx is often considered a purely cosmetic disorder, *Kelly and colleagues* reviewed autopsies and concluded that patients with PEx have a shorter life expectancy because of that surgical repair in adult is worthy and required more attention [19].

Furthermore, severity of PEx is associated with reduced pulmonary function and this correlate with our study as dyspnea was a significant complaint in both groups, mostly due to the reduction in lung volume secondary to compression, pain will limit chest wall expansion and arrhythmia will affect cardiac output specially in cases with supraventricular tachycardia or rapid AF [20].

There were asymptomatic cases with mild degree of deformity who were cosmetically unsatisfied without other manifestations, weren’t included in our study. Cosmetic interventions became available for those asymptomatic patients. Silicone implants and polyethylene implants are manipulated to cosmetically fix the deformity [21].

Open surgical reconstruction in adult age is recommended than Nuss procedure, as the flexibility of bone will be difficult to handle. Authors supported the same principle that significant Pectus deformities should undergo early surgical repair, preferably between one and 8 years of age as most of the deformity in these cases will be localized to the cartilage with minimal ribs affection. [22]. This was demonstrated between both groups as there were differences during operative and hospital course data with the advantage to perform the procedure as early as we can. The more rigid skeleton the more difficult the procedure will be (Table 6).

The majority of studies in Pectus deformity had been operated on during childhood; therefore there is limited published information about PEx deformity in adults. The most important point in Pectus correction is to achieve proper and long-term stability of the sternum following osteotomy and reposition of the sternum. Various techniques and supporting materials were used for this purpose [23].

Regarding the surgical procedure in our study; firstly longitudinal incision gave us excellent exposure to access all deformed costal cartilages in comparison to limited cartilage resection in other studies. Splitting of pectoral muscle laterally reduced postoperative pain and recovery time in comparison to muscle cutting. This technique provided excellent exposure to manage any bleeder either from intercostal arteries or internal mammary arteries. *Swanson* et al.*,* supported the same technique, longitudinal incision gave excellent exposure, accessibility to all deformed costal cartilages, allowing easy resection of cartilages with minimal complications, good healing and easy manipulation of rigid skeleton [21].

Perichondrium and periosteum are fibrous sheaths of vascular connective tissue surrounding the rib cartilage and bone segments, respectively. Reports in humans have indicated that both the costal cartilage and bone will regenerate over time when this connective tissue is left intact [3].

In our study, the use of one bar supporting sternum was satisfactory in all cases and its removal after 1 year was a shorter period without affection on the surgical result and decreased pain score. In other studies showed that, the majority of patients who underwent the Nuss procedure had their steel bar implants removed 2–3 years after the surgery which is longer period [21].

In this study, one case post-operative showed bar displacement which required surgical adjustment. The use of one bar facilitated its removal with low incidence of infection. Infection over the bar site was noticed in one case who required wound debridement without need to its removal. Other authors documented infection over the bar site in 4.4% of cases, which was slightly higher than our study [24].

In our study, complications were insignificant findings postoperatively, with insignificant differences between both groups, just numerical variation. Superficial and deep wound infection which occurred wasn’t significant finding. The predisposing factors were diabetes mellitus, obesity, and re-exploration. The percentage of failure of PEx repair was low (*n* = 1, 2.17%) in group II. The precipitating factors were combinations of obesity, severe and extremely severe HI, re-exploration, infection and those who didn’t follow postoperative instructions. The reported cases in other studies with deep sternal wound infection were 18.6%. *Shin* et al. reported that most postoperative wound infections can be treated conservatively with debridement [24].

By reviewing one of the meta-analysis studied the difference between Nuss procedure versus the Ravitch procedure with respect to overall complications, length of hospital stay, and time to ambulation, there was no difference. However, the rate of reoperation, postoperative haemothorax, and pneumothorax after the Nuss procedure were higher compared to the Ravitch procedure. No studies showed a difference in patient satisfaction [25, 26].

Regarding operative time, ICU stay and hospital admission in our study, showed similarity in comparison to other surgical techniques with significant differences between both groups, denoting longer time in group II than group I. This was explained by longer duration of procedure in elder age group with rigid skeleton and the liability of occurrence of complications in the same group [21].

Regarding postoperative instructions and follow up, they were important leading causes of success with low recurrence rate. Patient satisfaction was excellent in 77.9%, good in 17.4% and fair in 4.7% from total number of cases in both groups with insignificant difference in between both groups. According to the results, there were significant improvements of all preoperative symptoms as the grade of dyspnea, pain and rhythm disturbance. This is correlated with other studies encouraging Pectus repair in adults, one study which reviewed 317 patients who underwent a mid-term outpatient follow up. Patient satisfaction was excellent in 234 patients, good in 79 patients and fair in 4 patients with total improvement in patient’s symptoms. Surgical repair in adults improve and modify quality and life style of patients safely [23].

### Limitations

The prevalence of this deformity cannot be reflected from our study, large number of cases were excluded. There was difficulty to obtain data about early years of life; to detect any illness can affect bone growth. In addition to the lack of resources for follow up and family negligence. Long term results still need more studies.

## Conclusion

Surgical correction of Pectus Excavatum using open technique in adults had excellent postoperative outcome in the short term follow up that encourage performing the procedure for all cases. Long term results need longer period for follow up. Etiology and predisposing factors still need further research.

## Abbreviations

CDI: Cardiac deformity index
CT: Computed tomography
HI: Haller index
ICU: Intensive Care Unit
J-vac drain: Jackson-Pratt drain
LIMA: Left internal mammary artery
MRI: Magnetic resonant imaging
NSAID: Non-steroidal anti-inflammatory drugs
NYHA: New York Heart Association functional classification
PEx: Pectus excavatum
